# Architecture and Chemical Coding of the Inner and Outer Submucous Plexus in the Colon of Piglets

**DOI:** 10.1371/journal.pone.0133350

**Published:** 2015-07-31

**Authors:** Carola Petto, Gotthold Gäbel, Helga Pfannkuche

**Affiliations:** Institute of Veterinary Physiology, Faculty of Veterinary Medicine, Leipzig University, Leipzig, Germany; University of California, Los Angeles, UNITED STATES

## Abstract

In the porcine colon, the submucous plexus is divided into an inner submucous plexus (ISP) on the epithelial side and an outer submucous plexus (OSP) on the circular muscle side. Although both plexuses are probably involved in the regulation of epithelial functions, they might differ in function and neurochemical coding according to their localization. Therefore, we examined expression and co-localization of different neurotransmitters and neuronal markers in both plexuses as well as in neuronal fibres. Immunohistochemical staining was performed on wholemount preparations of ISP and OSP and on cryostat sections. Antibodies against choline acetyltransferase (ChAT), substance P (SP), somatostatin (SOM), neuropeptide Y (NPY), vasoactive intestinal peptide (VIP), neuronal nitric oxide synthase (nNOS) and the pan-neuronal markers Hu C/D and neuron specific enolase (NSE) were used. The ISP contained 1,380 ± 131 ganglia per cm^2^ and 122 ± 12 neurons per ganglion. In contrast, the OSP showed a wider meshwork (215 ± 33 ganglia per cm^2^) and smaller ganglia (57 ± 3 neurons per ganglion). In the ISP, 42% of all neurons expressed ChAT. About 66% of ChAT-positive neurons co-localized SP. A small number of ISP neurons expressed SOM. Chemical coding in the OSP was more complex. Besides the ChAT/±SP subpopulation (32% of all neurons), a nNOS-immunoreactive population (31%) was detected. Most nitrergic neurons were only immunoreactive for nNOS; 10% co-localized with VIP. A small subpopulation of OSP neurons was immunoreactive for ChAT/nNOS/±VIP. All types of neurotransmitters found in the ISP or OSP were also detected in neuronal fibres within the mucosa. We suppose that the cholinergic population in the ISP is involved in the control of epithelial functions. Regarding neurochemical coding, the OSP shares some similarities with the myenteric plexus. Because of its location and neurochemical characteristics, the OSP may be involved in controlling both the mucosa and circular muscle.

## Introduction

The enteric nervous system controls and modulates almost all functions of the gut [1]. The functions of the mucosal layer are primarily controlled by neurons located within the submucous plexus (SMP) [2, 3]. The submucous neurons influence the mucosal cells by releasing a cocktail of different inhibitory and/or excitatory neurotransmitters [3]. These neurotransmitters are expressed in distinct combinations within different functional classes of enteric neurons [4].

Until now, most data regarding the neurochemical coding of submucous neurons in the colon were derived from findings in small laboratory animals, such as mice and guinea pigs. These species possess a single-layered submucous plexus only [4]. In contrast, the SMP of large species (such as humans and pigs) is multilayered. The porcine colonic SMP consists of an inner submucous plexus (ISP), located close to the lamina muscularis mucosae, and an outer submucous plexus (OSP) located on the luminal side of the circular muscle layer [5]. In the human colon, a third intermediate plexus is located between the ISP and OSP [6, 7].

The partition of the SMP in large species is probably associated with a functional specialization of the subplexuses. This may also be reflected by the fact that the neurochemical codings of the inner and outer SMP differ from those of the submucous neurons of small laboratory species. Such differences have been already shown between the small intestines of pigs and guinea pigs [4, 8, 9]. However, data obtained from the small intestine of pigs cannot be easily transferred to the colon because these two gastrointestinal regions are functionally quite different. Therefore, the neuronal control might be adapted to the respective segment. Thus, to understand the neuronal control of colon-specific epithelial functions in large species, the determination of the neurochemical coding of the submucous neurons in the colon of such species is essential. Thus far, detailed studies determining the neurochemical codes of the two subplexuses in the porcine and human colon are scarce. Although some data regarding the neurochemical coding in the human SMP, derived from frozen sections of biopsy material, exist [10, 11], a detailed analysis of wholemount preparations from the submucous plexus is difficult due to the restricted availability of human tissues. Because of various similarities regarding gastrointestinal physiology and pathophysiology, the porcine enteric nervous system (ENS) has been previously suggested as a suitable model for the human ENS, in particular for the SMP [5, 8, 12–15]. Consequently, it seems reasonable to use the pig as a model for the determination of the neurochemical code in colonic submucous neurons.

Therefore, the aim of our study was the examination of the neurochemical coding in the inner and outer SMP of the proximal colon of three-week-old piglets. For this purpose, wholemount preparations of both submucous plexuses were examined using a multiplex-labelling immunofluorescence technique. The abundance of choline acetyltransferase (ChAT) as a marker for cholinergic neurons, somatostatin (SOM), neuropeptide Y (NPY), neuronal nitric oxide synthase (nNOS) as a marker for nitrergic neurons, substance P (SP) and vasoactive intestinal polypeptide (VIP) was analysed, and the co-localization of the different neurotransmitters and neuronal markers was detected. Additionally, cryostat sections of the colonic wall were labelled immunohistochemically to study the projections of nerve fibres within the mucosal and submucosal layers.

## Materials and Methods

### Tissue preparation

The experiments described in this study were carried out in strict accordance with the current German legislation covering the protection of animals (German animal welfare act and European guidelines directive 2010-63-EU). The experimental procedures were approved by the regional council of Saxony (permit number: T113/08) and all efforts were made to minimize suffering.

Specimens were obtained from 23 unweaned piglets (Deutsche Landrasse x Pietrain). Animals of both sexes, with an age of three weeks, were used. All animals were anaesthetized by intramuscular application of azaperone (2 mg/kg BW Stresnil, Janssen-CILAG GmbH, Neuss, Germany) and ketamine (20 mg/kg BW Ursotamin, Serumwerk Bernburg AG, Bernburg, Germany), followed by an intravenous application of thiopental (25 mg/kg BW Trapanal, Altana Pharma Deutschland GmbH, Konstanz, Germany). When no lid and corneal reflex could be provoked, the animals were exsanguinated by opening of the carotid arteries. The abdomen was opened and the proximal colon was removed. The colon contents were rinsed off by washes using ice-cold Krebs-Ringer solution of the following composition (in mM): 117 NaCl, 4.4 KCl, 1.2 MgCl_2_, 1.04 NaH_2_PO_4_, 25 NaHCO_3_, 2.5 CaCl_2_, 10 glucose and 1 μM nifedipine, pH 7.4, gassed with 5% CO_2_/95% O_2_.

To enhance the immunoreactivity of the neuropeptides NPY and VIP in submucous neurons, tissues were incubated with colchicine. For this purpose, washed tissues were pinned out in a Sylgard-covered petri dish and cultured for 24 h at 37°C, 5% CO_2_/95% O_2_ in culture medium (Dulbececco's modified Eagle's medium/F-12 Ham with 15 mM HEPES, 10% foetal calf serum, 100 U/ml penicillin, 100 μg/ml streptomycin, 50 μg/ml gentamicin, 5 μg/ml amphotericin B and 1 μM nifedipine) supplemented with 100 μM colchicine (all from Sigma Aldrich, Taufkirchen, Germany). During the culture period, the tissues were continuously agitated using a rocking tray. After organotypic culture, the specimens were fixed for 24 h at 4°C in 0.1 M phosphate buffer containing 4% paraformaldehyde and 0.2% picric acid. The fixed tissues were washed in 0.1 M phosphate buffer and stored in 0.1 M PBS containing 0.1% NaN_3_.

To evaluate possible effects of colchicine treatment, specimens (1 x 1 cm) of the proximal colon from three piglets were fixed immediately after tissue sampling in the same fixative mentioned above.

### Immunohistochemistry

For the immunohistochemistry of the ISP and OSP, wholemount preparations of the submucous plexus were obtained by removing the mucosal and muscle layers. These wholemount preparations were separated into the inner and outer parts of the submucous plexus. After the dissection, the tissues were pre-incubated and permeabilized for 1 h in PBS containing 4% horse serum (C.C. pro GmbH, Lörrach, Germany) and 0.5% Triton X-100 (Sigma-Aldrich). This solution was also used for the dilution of all antibodies. The tissues were incubated for 36 h at room temperature in a solution containing the primary antibodies (Table 1). After incubation with the primary antibodies, the specimens were washed three times in PBS and incubated for 5 hours in solution containing the secondary antibodies (Table 2). Finally, the specimens were washed in PBS, mounted on slides and covered with a solution of NaHCO_3_/Na_2_CO_3_ (0.5 M, pH 7.0) containing 0.1% NaN_3_ and 80% glycerol.

**Table 1:** List of primary antibodies.

Primary antibodies				
Antigen	Host	Dilution	Reference number	Supplier
ChAT	Rabbit	1: 1000	P3YEB	[16]
Neuropeptide Y	Rabbit	1: 1000	T-4453	Peninsula, USA
nNOS Type I	Mouse	1: 40	610309	BD Transduction Laboratories, USA
Somatostatin	Rabbit	1: 1000	T-4102	Peninsula, USA
Substance P	Rat	1: 1000	10-S15A	Fitzgerald, USA
Substance P	Rat	1: 1000	ab7340	Abcam, UK
VIP	Guinea pig	1: 1000	T-5030	Peninsula, USA
Hu C/D	Mouse	1: 200	A-21271	Molecular Probes, MoBiTec, Germany
NSE	Rabbit	1: 500	16625	Polysciences, USA

**Table 2:** List of secondary antibodies.

Secondary antibodies (all from Jackson ImmunoResearch, Dianova, Germany)		
	Dilution	Reference number
Biotin-SP-conjugated donkey anti-guinea pig IgG	1: 50	706-065-148
Dylight 405-conjugated streptavidin	1: 50	016-470-084
AMCA-conjugated streptavidin	1: 50	016-150-084
Cy 2-conjugated donkey anti-mouse IgG	1: 200	715-225-151
Cy 3-conjugated donkey anti-rabbit IgG	1: 500	711-165-152
Cy 5-conjugated donkey anti-rat IgG	1: 500	712-175-153
Dylight 649-conjugated donkey anti-rat IgG	1: 500	712-495-153

To determine the proportion of distinct, neurochemically-defined subpopulations in the ISP and OSP in four out of 18 animals, three combinations of four primary antibodies, raised in different host species, were used (Table 3). For quantification of the subpopulations, microscopic images of the ganglia were taken. Furthermore, the positions of the ganglia in the specimens were recorded to enable their subsequent retrieval. After examination, the preparations were unmounted and washed three times in PBS to remove the mounting medium. Thereafter, the specimens were stained a second time with an antibody against pan-neuronal protein Hu C/D, and the previously analysed ganglia were re-examined. The Hu C/D antigen is exclusively present in neurons [17] and is useful for the determination of the total number of neurons per ganglion in the investigated tissues.

**Table 3:** Antibody staining combinations for determining the neurochemical subpopulations.

Combination A	Combination B	Combination C
Rabbit anti—ChAT	Rabbit anti—SOM	Rabbit anti—NPY
Mouse anti—nNOS	Mouse anti—nNOS	Mouse anti—nNOS
Rat anti—SP	Rat anti—SP	Rat anti—SP
Guinea pig anti—VIP	Guinea pig anti—VIP	Guinea pig anti—VIP

To study the presence of nerve fibres within the mucosal and submucosal layer, immunohistochemical labelling was performed on cryostat sections of the colonic wall obtained from three piglets. The fixed tissues were prepared for the cryostat sections by incubating them overnight at 4°C in PBS containing 0.1% NaN3 and 30% sucrose. Transversal sections (12 μm) were cut by a cryostat, mounted on superfrost^++^ slides and stored at 20°C. After washing and preincubation for 1 h in PBS containing 4% horse serum and 0.5% Triton X-100, the sections were incubated with the same primary antibodies used for wholemount preparations for 15 h at room temperature. Neuron specific enolase (NSE) was used as a general neuronal marker (Table 1). NSE is expressed in neuronal somata as well as in nerve fibres.

With each antibody combination at least six sections per piglet were stained. After incubation with the primary antibodies, the sections were washed three times in PBS and incubated for 2.5 hours in solution containing the secondary antibodies (Table 2). Finally, the sections were washed in PBS and covered with a solution of NaHCO_3_/Na_2_CO_3_ (0.5 M, pH 7.0) containing 0.1% NaN_3_ and 80% glycerol.

#### NADPH staining

The NADPH-diaphorase reaction was used to detect nitrergic somata and nerve fibres in wholemount preparations and in cryostat sections. To visualize NADPH-diaphorase, tissues were incubated at 37°C in 0.1 M phosphate buffer (pH 8) containing 0.5% Triton X, ß-NADPH-D (0.5 mg/10 ml, Sigma-Aldrich) and 4-Nitrotetrazoliumblau (1mg/10ml, Sigma-Aldrich). The reaction was stopped after 2 h by several rinses with PBS.

#### Specificity of primary and secondary antibodies

The specificity of the antibodies against SOM, NPY, nNOS, SP and VIP (all from Bachem, Heidelberg, Germany) was tested by adsorption of the respective diluted antibodies with 1 μM of SOM, NPY, SP or VIP for 24 h at room temperature prior to their application. Applying the pre-adsorbed antibodies to the tissues did not result in any positive staining of the preparations. The specificity of the ChAT antibody P3YEB [16] was shown previously by Hens et al. (2000) [18]. The specificity of the mouse anti-nNOS antibody was determined by detecting the NADPH-diaphorase reaction in tissues stained first against nNOS. The mouse anti-nNOS antibody and the NADPH-diaphorase reaction marked identical neurons. To prove the specificity of the secondary antibodies, they were applied without using the primary antibodies. No staining was seen after omitting the primary antibodies.

### Analysis of neurochemical coding

Specimens were examined using an epifluorescence microscope (IX50, Olympus, Japan) with a black and white video camera (F-view, Olympus, Hamburg, Germany) attached to an image analysis system (cell^F, Olympus, Hamburg, Germany).

For all markers, the number of neurons per ganglion was counted in 20 randomly chosen ganglia in each specimen. For each preparation, median values of the numbers of immunoreactive neurons per ganglion were calculated. These median values were used to calculate the mean for all animals. Results are expressed as means ± standard error of the mean (SEM; *n* = number of preparations, *N* = number of animals). To obtain the proportion of neurochemically defined subpopulations, the number of neurons showing immunoreactivity for a distinct marker was correlated to the total number of neurons of the respective ganglion.

The numbers of ganglia per cm^2^ were calculated from cell^F images of the anti-Hu C/D stained specimens in a 100-fold magnification. An image covers an area of 0.037 cm^2^. All ganglia included in this area and all ganglia touching the left or the bottom margin were counted. Two images per specimen were analysed. The mean value was calculated from the specimens of the four animals, which were examined for the proportions of the subpopulations.

The Mann-Whitney rank-sum test was used for statistical evaluation of the total numbers of neurons per ganglia of the ISP and OSP and to compare colchicine treated and non-treated tissue preparations. The proportions of subpopulations were compared between the ISP and OSP using a two-way repeated-measurements analysis of variance, with a subsequent multiple comparison procedure (Student-Newman-Keuls test). Statistical tests were performed using SigmaStat 3.5 (SPSS Science, Chicago, IL). Differences were considered as statistically significant at values of *P* < 0.05.

## Results

### General architecture of the SMP

**Fig 1:**
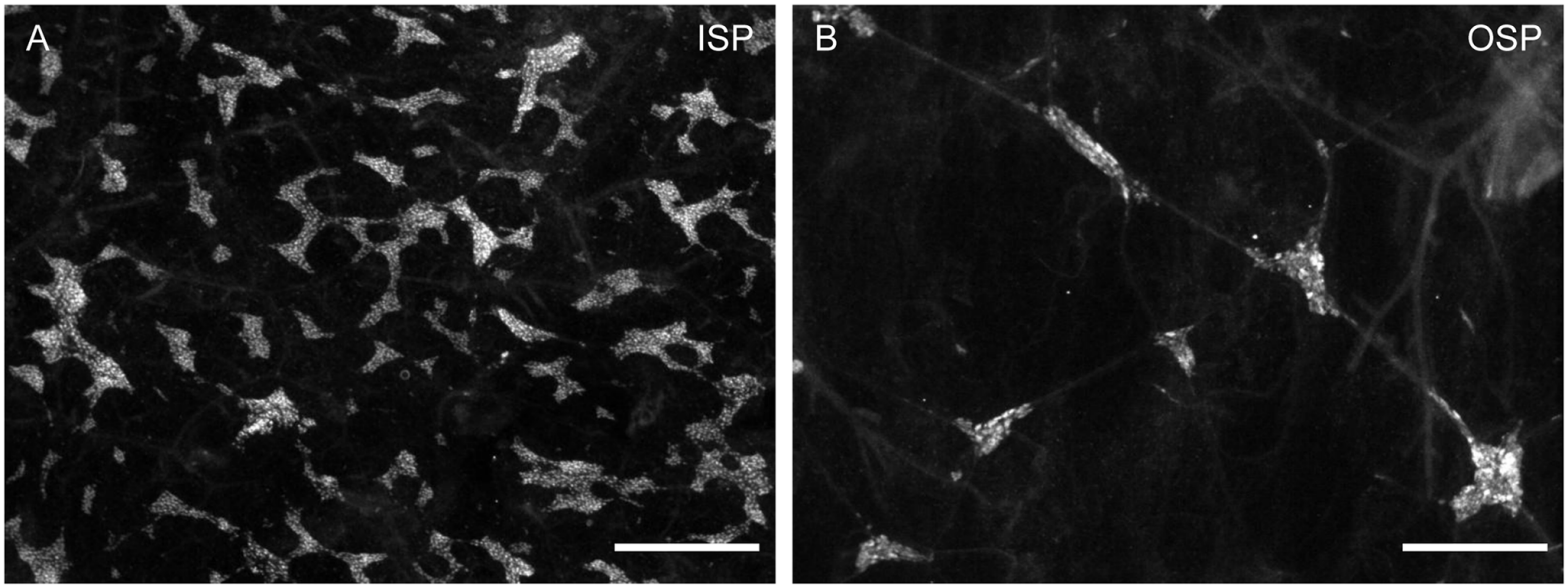
General architecture of the ISP (A) and OSP (B) in the porcine colon. Specimens were immunohistochemically stained for the pan-neuronal marker Hu C/D. The ISP consists of a dense network composed of ganglia that are highly variable in size and shape, whereas the OSP comprises a wider meshwork consisting of significantly fewer ganglia per cm^2^ and significantly smaller ganglia than the ISP. Scale bar = 0.5 mm.

The ISP and OSP differed considerably in their morphology and neuronal density. The inner plexus was a dense network composed of ganglia that were highly variable in size and shape (Fig 1A). The ISP contained 1,380 ± 131 ganglia per cm^2^ and 122 ± 12 neurons per ganglion. In contrast to the ISP, the OSP showed a wider meshwork (Fig 1B) consisting of significantly fewer ganglia per cm^2^ and significantly smaller ganglia (215 ± 33 ganglia per cm^2^; n = 12; N = 4 and 57 ± 3 neurons per ganglion, n = 16; N = 8, paired t-test, *P* < 0.001)

### Total numbers of immunoreactive neurons

Quantification of neuronal subpopulations described in this study is based on the number of immunoreactive somata.

**Fig 2:**
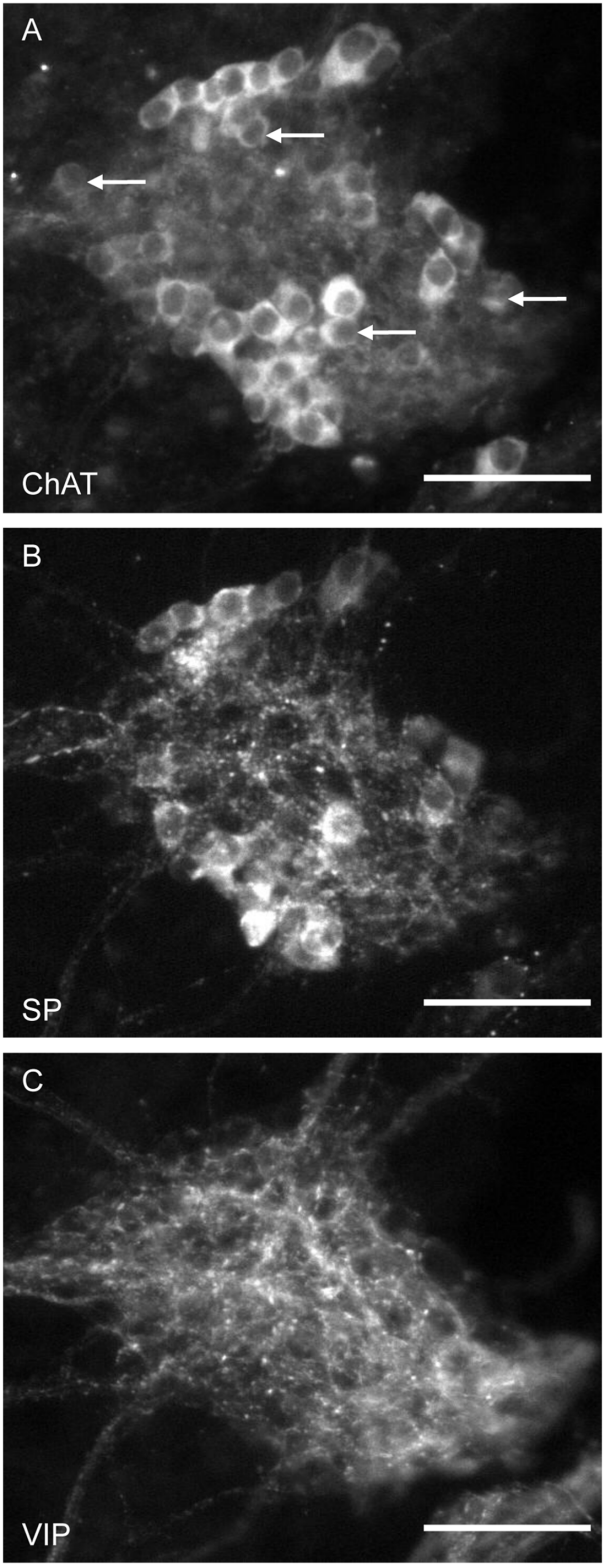
Multi-labelled ganglion in the ISP of the porcine colon. The specimen was immunohistochemically stained for ChAT, SP and VIP. Immunoreactive neurons were found for ChAT (A) and SP (B). For VIP (C), only immunoreactive fibres were found in ISP ganglia. Arrows indicate neurons immunoreactive only for ChAT. All other immunoreactive neurons co-localized with ChAT and SP. Scale bar = 50 μm.

In the neuronal somata of the ISP, mainly ChAT and SP immunoreactivity were detected (Fig 2A and 2B, Table 4). About one-third of all neurons were ChAT positive. These neurons are subsequently referred to as cholinergic neurons. Immunoreactivity for SP was found in about one-quarter of all neurons. Neurons expressing nNOS (subsequently referred to as nitrergic neurons) were sparse and were found only in the ISP of two out of 12 animals.

**Table 4:** Absolute numbers of immunoreactive neurons per ganglion in the ISP and OSP.

Antigen	ISP	OSP	significant difference between ISP and OSP
	(n = 18; N = 8)	(n = 25; N = 12)	*P*
Hu C/D	122.6 ± 12.0	56.8 ± 2.9	< 0.001
ChAT	48.5 ± 7.4	19.2 ± 2.3	< 0.05
NPY	0.2 ± 0.2	2.1 ± 1.2	n.s.
nNOS	0.2 ± 0.2	16.1 ± 1.0	< 0.001
SOM	0.9 ± 0.2	3.5 ± 0.9	< 0.05
SP	32.7 ± 2.8	11.9 ± 1.0	< 0.001
VIP	3.7 ± 2.6	6.3 ± 1.7	< 0.05

means ± SEM, n = number of tissues, N = number of animals. n.s. = non-significant

In the vast majority of ISP specimens, immunoreactivity for VIP was limited to nerve fibres (Fig 2C). VIP-immunoreactive somata were observed only in the ISP of three out of 12 animals. In almost every ganglion in the ISP, one or two SOM-expressing neuronal somata were detected. Staining against NPY revealed a dense network of nerve fibres surrounding blood vessels running between both plexuses (S1 Fig). NPY-immunoreactive somata were not present in the ISP

**Fig 3:**
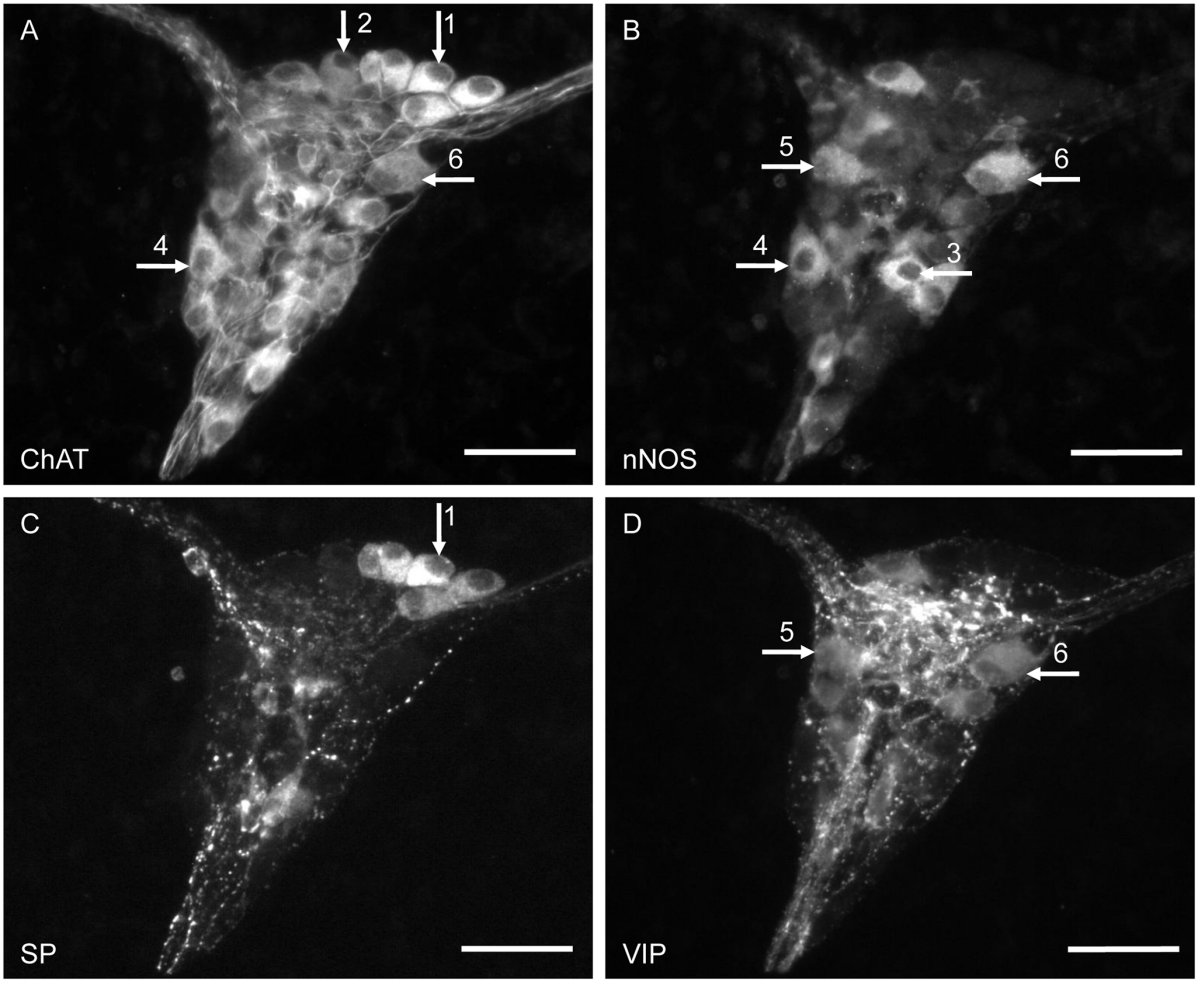
Multi-labelled ganglion in the OSP of the porcine colon. The specimen was immunohistochemically stained for ChAT, nNOS, SP and VIP. The neurochemical coding detectable in the OSP showed a higher variability compared to the ISP. Immunoreactive neurons were found for ChAT (A), nNOS (B), SP (C) and VIP (D). Arrows indicate neurons of the following subpopulations/coding: 1) ChAT/SP, 2) ChAT/-, 3) nNOS/-, 4) ChAT/nNOS, 5) nNOS/VIP and 6) ChAT/nNOS/VIP. Scale bar = 50 μm.

In contrast to the ISP, all neurotransmitters or neuronal markers examined could be detected in the neuronal somata of the OSP (Table 4, Fig 3). The highest numbers of immunoreactive neurons were found for ChAT, SP and nNOS. The higher content of nNOS- and of VIP-expressing somata, as well as the presence of NPY in neuronal somata in the OSP, were the most striking differences between the ISP and OSP (Fig 3B and 3D, Table 4).

### Neurochemically identified subpopulations in the ISP

**Fig 4:**
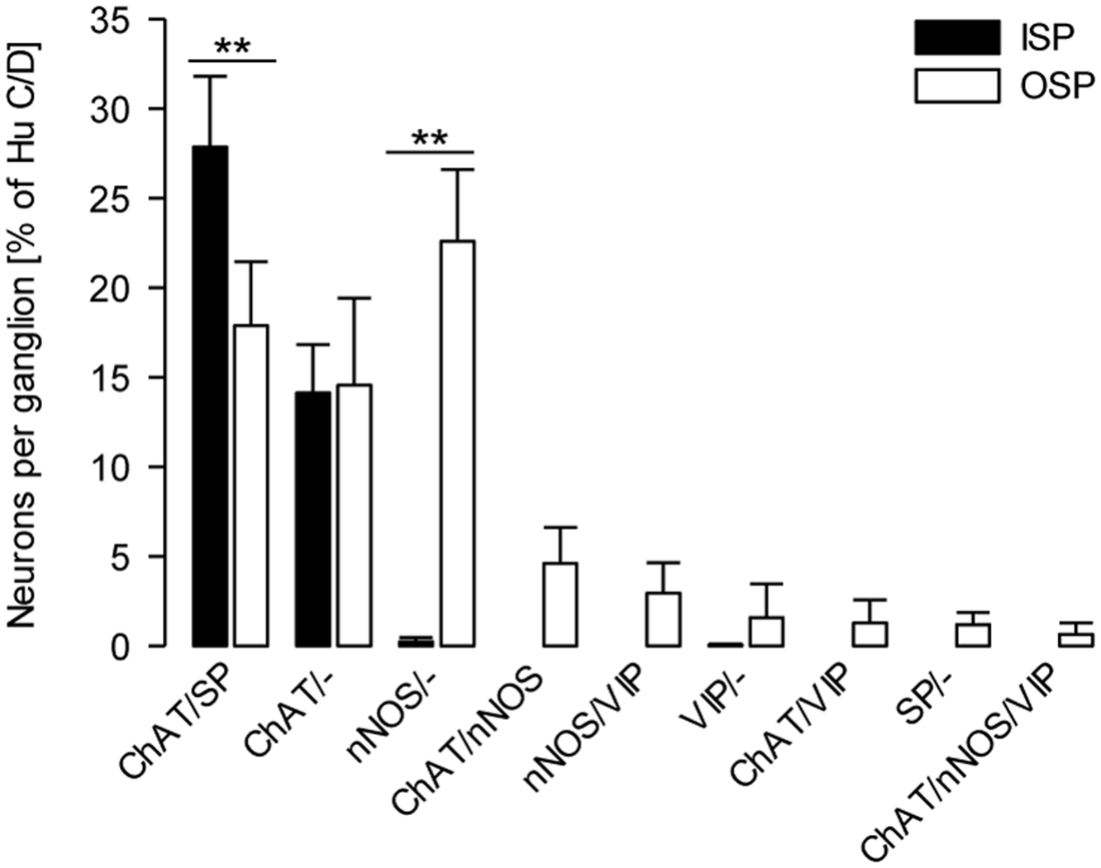
Relative distribution of neurochemically-defined subpopulations in the submucous plexuses of the porcine colon. Specimens of the ISP and OSP were immunohistochemically stained for ChAT, nNOS, SP, VIP and the pan-neuronal marker Hu C/D. The numbers of neurons per ganglion for the respective subpopulations are presented as mean percentage ± SEM of Hu C/D-immunoreactive neurons per ganglion. ISP versus OSP ** *P* ≤ 0.001; N = 4, n = 12. See S1 Table for significant differences between subpopulations within the ISP or OSP.

In the ISP, 43% of all Hu C/D-immunoreactive neuronal somata expressed at least one additional neuronal marker or neurotransmitter. This was mainly ChAT (97% of the multi-labelled somata). About 66% of the cholinergic neurons also expressed SP (ChAT/SP; Fig 4). Neurons immunoreactive for SP only could not be detected, but SP-positive neurons always co-localized ChAT. No neurotransmitter other than SP was co-localized with ChAT.

Despite the main ChAT/SP-immunoreactive population, some additional small populations could be identified in the ISP. In one out of four animals, we found less than 0.25% nNOS- and VIP-immunoreactive somata in the ISP, which were not co-localized with each other or with ChAT. We observed SOM-immunoreactive neuronal somata in all preparations examined, although in very low numbers (0.85% of all Hu C/D-labelled neurons).

### Neurochemically identified subpopulations in the OSP

The neurochemical coding of the OSP was more complex than that of the ISP. In the OSP, we classified 72% of all neurons by their immunoreactivity for ChAT or NOS.

39% of all neurons were cholinergic (Fig 4). About 38% of cholinergic neurons were exclusively immunoreactive for ChAT (ChAT/-). Forty-six percent of the cholinergic neurons also expressed SP (ChAT/SP). The co-localization with ChAT accounted for the majority of SP-positive somata (94%). Only 6% of SP-immunoreactive neurons displayed no co-localization with ChAT or any other neurotransmitter examined (SP/-).

Expression of nNOS was found in 31% of all neurons from the OSP. The nitrergic neurons were mainly immunoreactive for nNOS only (74% of all nitrergic neurons). About 10% of the nitrergic neurons co-localized with VIP (nNOS/VIP). Co-localization of nNOS and VIP accounted for 56% of all VIP-immunoreactive neurons. The remaining VIP-expressing neurons either co-localized ChAT (VIP/ChAT) or did not co-localize any other neurotransmitter (VIP/-). However, the proportion of VIP-positive somata in the total population of neurons in the OSP was low (6% of all neurons).

In addition to neurons that were clearly assigned as cholinergic or nitrergic, a third group of neurons could be identified. These neurons showed an intermediate phenotype by expressing immunoreactivity for ChAT and nNOS (4.6% of all neurons). About 12% of all ChAT-expressing and 15% of all nNOS-expressing neurons displayed this ChAT/nNOS co-localization. Some of the ChAT/nNOS-immunoreactive neurons also expressed VIP (ChAT/nNOS/VIP: 2% of all ChAT, 2% of all nNOS-expressing and 11% of all VIP-expressing neurons). In two out of four animals, a few OSP ganglia contained a low number of neuronal somata immunoreactive for NPY (0.23% of all neurons), although NPY-positive fibres were found in all preparations examined. The number of SOM-immunoreactive somata in the OSP (4.3% of all neurons) was higher compared to the ISP (0.85% of all neurons). As in the ISP, no additional neurotransmitter was co-localized with SOM. However, as mentioned above, a co-localization of SOM and ChAT cannot be excluded.

### Effect of colchicine treatment

All results regarding the neurochemical coding of neuronal somata were obtained from wholemount preparations treated with colchicine. However, colchicine may not only enhance the presence of neuropeptides within the neuronal somata but also alter the proportion of ChAT or NOS-immunoreactive neurons. To check colchicine effects on the proportions of cholinergic and nitrergic neuronal subpopulations, wholemount preparations not treated with colchicine were analysed and compared to colchicine treated specimens (Table 5). We found no differences regarding the proportion of ChAT, nNOS, or SOM-immunoreactive somata between colchicine treated and untreated specimens. The proportion of SP-immunoreactive somata was slightly decreased in colchicine treated tissues of the ISP. Immunoreactivity for NPY was not visible in neuronal somata in untreated tissues. VIP expression occurred only occasionally without colchicine incubation in nerve cell bodies.

**Table 5:** Proportions of neuronal populations in tissues incubated with colchicine and untreated tissues.

Antigen	ISP (%)		OSP (%)	
	colchicine treated	non treated	colchicine treated	non treated
	(n = 12; N = 4)	(n = 9; N = 3)	(n = 12; N = 4)	(n = 9; N = 3)
ChAT	42.0 ± 4.5	39.8 ± 3.9	38.3 ± 5.7	35.9 ± 3.0
nNOS	0.2 ± 0.2	0.0 ± 0.0	30.2 ± 2.2	32.3 ± 2.7
NPY	0.0 ± 0.0	0.0 ± 0.0	0.2 ± 0.2	0.0 ± 0.0
SOM	0.9 ± 0.3	0.4 ± 0.2	4.3 ± 2.2	5.2 ± 0.9
SP	29.0 ± 1.5*	35.0 ± 1.6	21.1 ± 2.5	21.8 ± 1.4
VIP	0.1 ± 0.1	0.0 ± 0.0	5.8 ± 2.0	1.0 ± 0.8

means ± SEM, n = number of tissues, N = number of animals. * = significant difference (p < 0.05) between treated and untreated tissues

### Projections of neuronal fibres

**Fig 5:**
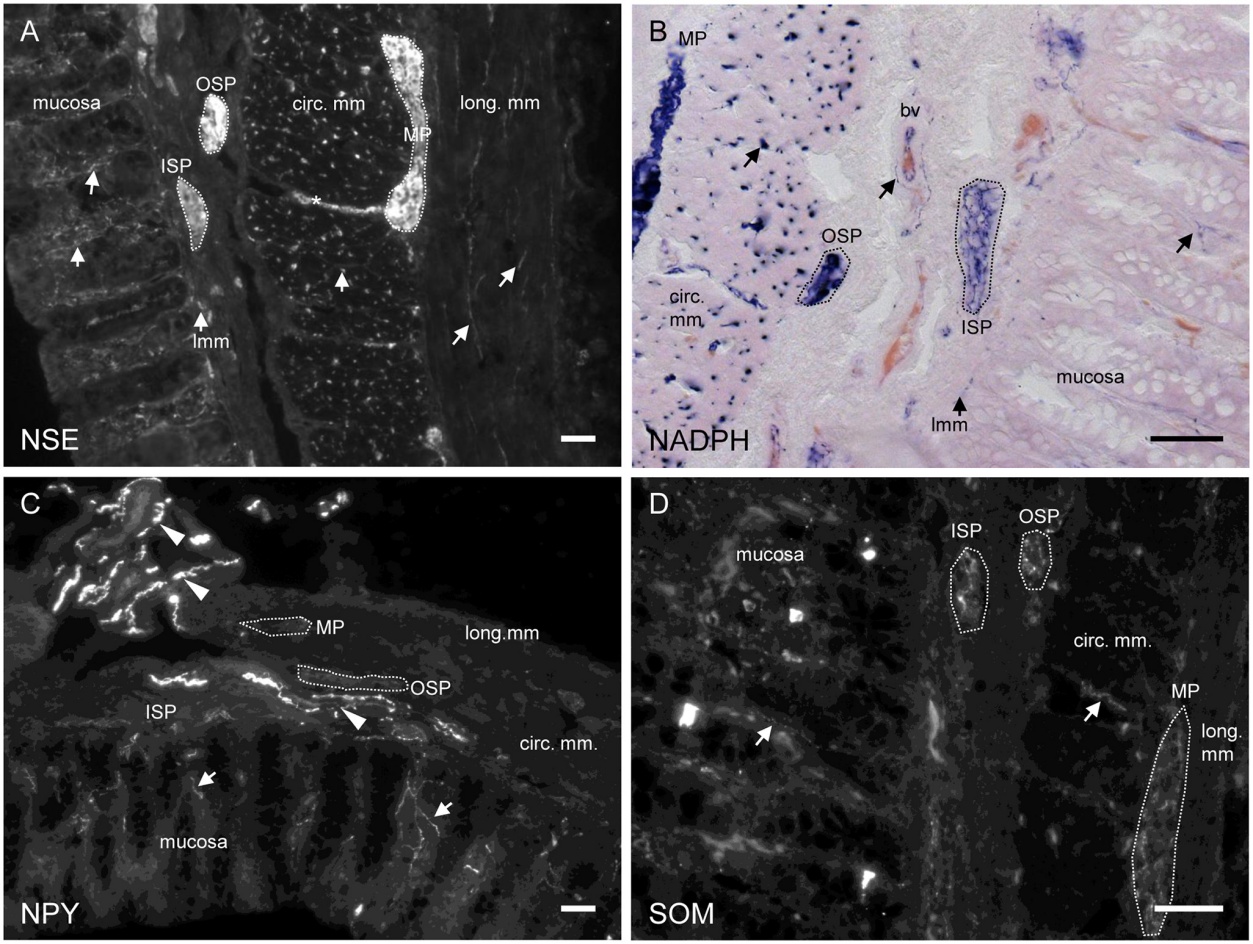
Transversal sections of piglet proximal colon stained for NSE, NADPH, NPY or SOM. (A) NSE staining revealed nerve fibres in all tissue layers (indicated by arrows). The asterisk marks a fibre bundle projecting from a myenteric ganglion presumably to the OSP. The dotted lines indicate ganglia within the ISP, OSP and MP (also in B, C and D). (B) Nitrergic neuronal fibres (in dark blue, marked by arrows) innervating the circular muscle layer, the mucosa and surrounding blood vessels (bv). (C) NPY staining strongly labelled fibers running with blood vessels (arrowheads). Additionally, NPY-immunoreactive fibres were found in the mucosa (arrows). (D) SOM-immunoreactive fibres were mainly detected within ganglia of the ISP, OSP or MP. The arrows indicate fibres running in the mucosa or the circular muscle layer. Scale bar = 50 μm.

Using the general neuronal marker NSE we found nerve fibres within all tissue layers of the sections i.e. mucosa, lamina muscularis mucosae, SMP, myenteric plexus (MP), circular and longitudinal muscle layer as well as around blood vessels (Fig 5A). Staining of fibres was strongest within the MP and the circular muscle layer.

**Fig 6:**
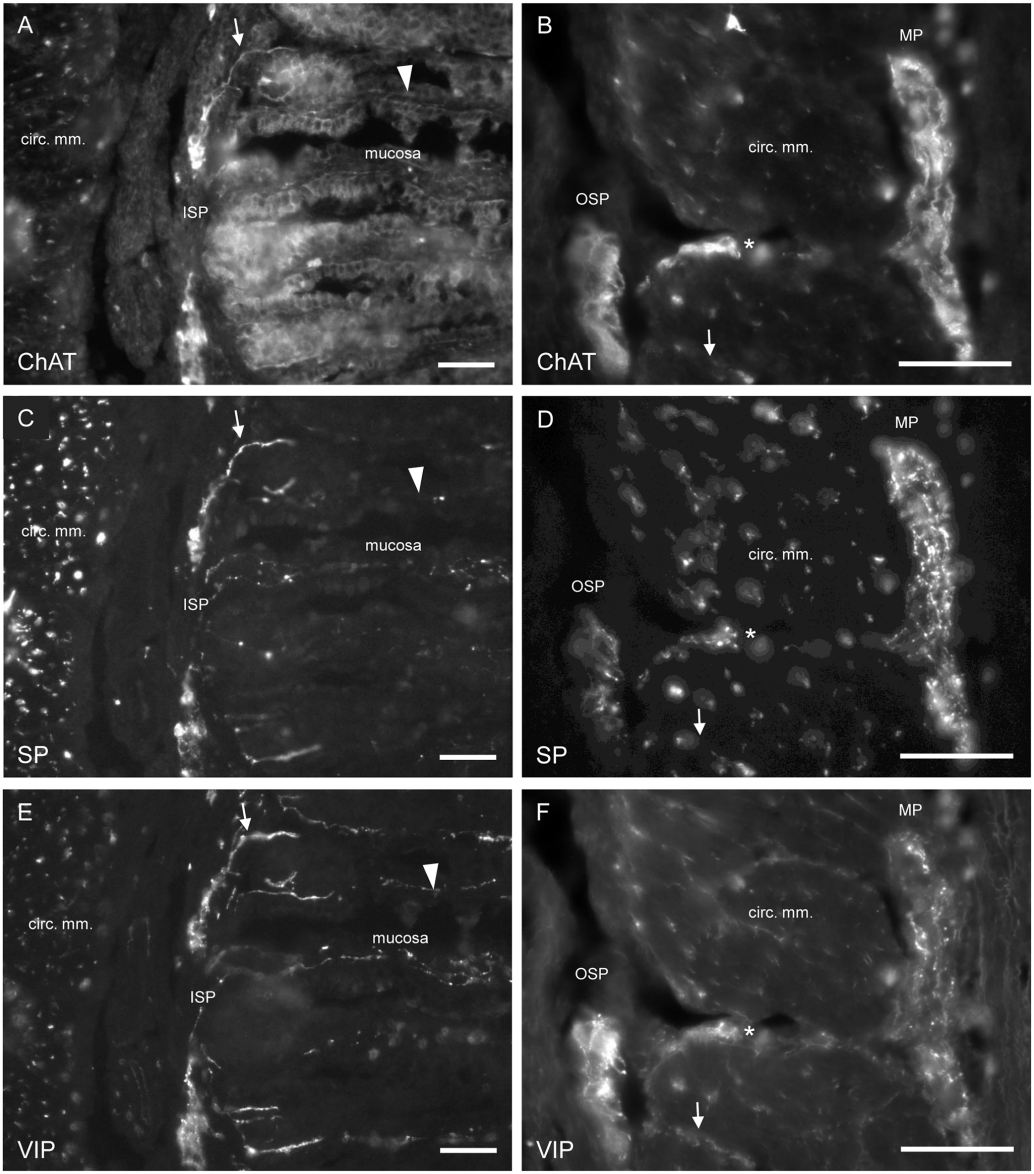
Immunohistohemical staining of transversal colonic sections labelled for ChAT (A, B), SP (C, D) and VIP (E, F). The same detail of one section is shown in (A), (C) and (E) and in (B), (D) and (F), respectively. Neuronal fibres projecting from an ISP ganglion into the mucosa are immunoreactive for ChAT, SP and VIP (indicated by an arrow in A, C and E). The arrowhead marks a nerve fibre (bundle) not immunoreactive for ChAT (A), but weakly for SP (C) and strongly for VIP (E). (B), (D) and (F) show a nerve fibre bundle (marked by an asterisk) projecting between an OSP ganglion and a ganglion of the MP, expressing immunoreactivity for ChAT (B), SP (D) and VIP (F). Additionally, some fibres (arrow) running from OSP into the circular muscle layer are visible. Scale bar = 50 μm.

Staining against ChAT, SP and VIP revealed neuronal fibres in almost all layers. Only in the small area around blood vessels no fibres immunoreactive for SP or ChAT could be detected. Regarding the innervation of the mucosal layer, immunoreactivity for ChAT, SP or VIP occurred usually within the same fibre bundles projecting from ISP ganglia into the mucosa (Fig 6A, 6C and 6E). Single fibres immunoreactive only for one or two of these substances were also found occasionally (Fig 6A, 6C and 6E). Projections from the OSP to the mucosa were not visible but fibres running between OSP ganglia and from OSP ganglia into the circular muscle layer could be observed (Fig 6B, 6D and 6F). Additionally, some neuronal fibre bundles ran between OSP and MP ganglia (Fig 6B, 6D and 6F).

Immunostaining against NOS resulted in only weak labelling of neuronal fibres. NADPH staining revealed neuronal fibres around blood vessels, within the circular muscle layer, MP, ISP, OSP, the *lamina muscularis mucosae* and single projections also within the mucosa (Fig 5B). In the longitudinal muscle layer NADPH positive fibres were not visible. NPY-immunoreactive neuronal fibres were primarily present within ganglia of the OSP and MP (Fig 5C). Additionally, NPY expression was seen in fibres projecting to the mucosa and surrounding blood vessels (Fig 5C). Immunoreactivity for NPY could not be detected in ganglia of the ISP and in the circular and longitudinal muscle layers. Staining against SOM revealed a strong signal in all ganglia examined, independent of the plexus layer. Additionally, we found few neuronal fibres in all muscle layers and within the mucosa (Fig 5D).

## Discussion

In the present study, we examined the general architecture and the neurochemical coding of the two submucous plexuses in the proximal colon of piglets. We found that the ISP and OSP differed considerably a) in ganglion size and the number of ganglia per cm^2^ and b) in their neurochemically-identified subpopulations.

### General architecture of the ISP and OSP

The OSP displayed a wider meshwork with a lower number of ganglia per cm^2^ than the ISP in the porcine colon. This observation is in accordance with findings from the porcine ileum and the human colon, where the ISP also forms a denser network than the OSP [7, 19–21].

Not only the number of ganglia per cm^2^, but also the number of neurons per ganglion, varied between the two submucous plexuses. We observed twice as many neurons per ganglion in the ISP (122±12) compared to the OSP (57±3). In wholemount preparations of the ileum of 12 week-old piglets, Kapp et al. (2006) found that the ISP ganglia contained only 1.5 as many neurons (112 neurons per ganglion) as the OSP (82 neurons per ganglion).

The considerable difference in ganglion size between the ISP and OSP could be a species-specific phenomenon of the porcine colon. In frozen sections of the human colon, differences in the size of ganglia between the ISP and OSP were not as large as in the porcine colon. Wedel et al. (2002) identified on average 2.4 neurons per ganglion in human colonic OSP, 1.7 neurons per ganglion in the intermediate submucous plexus and 1.6 neurons per ganglion in the ISP.

Despite the lack of strong differences in ganglion size between the ISP and OSP, Wedel et al. (2002) described significantly lower numbers of neurons per ganglion in the human colon compared to the porcine colon. This might be due to interspecies differences. However, differences in the methods of preparation and/or age of the subjects studied also have to be taken into account. In general, the number of neurons per ganglion determined in sections seems to be consistently lower than that in wholemounts [21–23]. Regarding the age of subjects, the mean age of patients was 64 years in the study of Wedel et al. (2002). The differences between our results and those of Wedel et al. (2002) might be at least partially explained by the significantly higher age of the subjects examined in the latter study. Similar to the central nervous system (CNS), there is a strong decline in the number of neurons during the maturation of the porcine and human ENSs [24, 25].

### Neurochemical coding of the ISP

The distribution of neurochemically-defined neuronal populations differed between the two submucous plexuses. In the ISP, we detected, almost exclusively, ChAT-immunoreactive and SP-immunoreactive neurons, and only a small but consistent number of SOM-positive somata. VIP- or nNOS-immunoreactive neurons were extremely scarce. NPY-positive somata were totally absent from the ISP.

The cholinergic neurons in the colonic ISP might have various functions. It is likely that at least some of the cholinergic neurons directly project to the mucosa and might serve as secretomotor neurons. This assumption is based on the observation that various ChAT-immunoreactive nerve fibres projected from the ISP ganglia to the mucosal layer (Fig 6). Additional evidence for a secretomotor function is provided by tracing studies conducted by Hens et al. (2000) in the porcine small intestine. The study showed that more than 70% of neurons projecting to the epithelium were located within the ISP [18]. Functional studies [26–28] also suggest a role as secretomotor neurons for the cholinergic subpopulation showing that ACh exhibits a strong prosecretory effect in the porcine gastrointestinal tract, including the proximal colon.

A large number of the cholinergic somata in the ISP co-localized with SP, and numerous SP positive fibres were found within the mucosa, which at least partly originated from ISP ganglia. Like ACh, SP is a strong secretagogue in the gastrointestinal tract of pigs [28–33]. Consequently, the ChAT/SP-immunoreactive neurons we detected might belong to the population of cholinergic secretomotor neurons projecting to the mucosa. Additionally, in the porcine small intestine, ChAT/SP-immunoreactive mucosally projecting neurons have been classified as secretomotor neurons by Brown and Timmermans (2004).

Besides secretomotor effects, ChAT/SP-immunoreactive neurons might also act as interneurons localized within the ISP. We observed ChAT and SP-immunoreactive fibres not only projecting into the mucosa, but also within the ganglia of the ISP, OSP and MP. However, in the preparations we examined, direct neuronal connections between the ISP and the other plexuses could not be found. Nevertheless, evidence for SP as a modulator of other submucous neurons at least inside the ISP is provided by various functional studies, showing that SP not only directly acts on colonocytes, but also exhibits a prosecretory action via the activation of other submucous neurons [28, 34–37].

### Neurochemical coding of the OSP

The neurochemical coding of the neurons in the OSP showed a higher variability compared to the neurons of the ISP. Most ganglia in the OSP contained neurons that were immunoreactive for all neurotransmitters or neuronal markers studied.

A large population (39%) of OSP neurons displayed a cholinergic phenotype. Because of their localization between the epithelium and the circular muscle layer, these neurons might be involved in the control of both epithelial secretion and motility. The latter function seems to be more reasonable since we found no projections from the OSP to the mucosa, independent of the neurotransmitter examined. Additionally, the OSP ganglia were situated in very close contact to the circular muscle layer and we observed neuronal fibres projecting from the OSP ganglia into the circular muscle. Nevertheless, it cannot be excluded that at least a portion of the cholinergic neurons in the OSP probably functions as secretomotor neurons. This might be concluded because a) in the porcine small intestine, about 10% of mucosally-projecting neurons are located in the OSP, and b) more than 80% of these neurons are cholinergic [18].

Apart from the putative epithelial projections of OSP neurons, a substantial number of OSP neurons might innervate the adjacent muscle layers [5, 18, 38, 39]. In the circular muscle layer we identified neuronal fibres immunoreactive for ChAT, SP, NOS, VIP or SOM. However, these fibres may not only originate from OSP ganglia but also from ganglia located within the MP. Regarding projections from the OSP into the circular muscle layer, studies by Sanders et al. (1986) in the canine proximal colon provided evidence of an excitatory innervation of the circular muscle layer by cholinergic motor neurons located in the submucous plexus [40]. Similarly, Furness et al. (1990) found structural evidence for submucosal neurons immunoreactive for SP and VIP projecting to the circular muscle layer in the colon and small intestine of the dog [41]. Hens et al. (2002) described that in the longitudinal muscle layer is innervated not only by the MP, but also by ISP and OSP neurons in the porcine small intestine [42]. If smooth muscle layers are innervated by OSP neurons, ChAT/±SP might function as excitatory motor neurons innervating the circular or longitudinal muscle layer. This function is supported by the fact that the application of both ACh and SP leads to contractions of the circular muscle layer in all gastrointestinal regions examined thus far [43–45].

Besides the large cholinergic neuronal population, an almost similar sized population (31%) of nitrergic neurons was observed in the OSP. This predominance of nitrergic neurons observed in the OSP compared to the ISP is in accordance with the findings of Barbiers et al. (1993), who detected one or two nitrergic neurons per ganglion in the ISP but 15 nNOS-positive neurons per ganglion in the OSP [46] of the porcine colon. In the human colon, nitrergic neurons were rare in the ISP, but numerous in the OSP [23]. Seventy-four percent of the nitrergic neurons in the porcine OSP did not co-localize any other marker used in the present study. A small population (10% of the nitrergic neurons) co-expressed VIP. According to the classification by Brown and Timmermans (2004), both subpopulations (nNOS/- and nNOS/VIP) could be assumed to be inhibitory motor neurons. The relatively high proportion of motor neurons would highlight the contribution of the OSP in the regulation of motility [47]. However, it cannot be excluded that a subpopulation of nitrergic neurons classified in this study contributes to the regulation of mucosal secretion. nNOS- and VIP-immunoreactive fibres are present in the mucosa of the porcine proximal colon [28]. Furthermore, functional evidence comes from studies of Pfannkuche et al. (2011) and Gäbel et al. (2003), who demonstrated that nitric oxide, as well as VIP, causes a strong secretory response in the proximal colon of piglets and adult pigs, respectively. Whether the putative nitrergic secretomotor neurons in the OSP express the code nNOS/-, nNOS/VIP or belong to the “intermediate” subpopulation with the neurochemical code nNOS/ChAT/VIP remains speculative.

In the MP of the porcine ileum, ChAT/nNOS-co-localizing neurons were classified as descending interneurons [48]. However, as mentioned above, ACh is a strong prosecretory substance, like nitric oxide and VIP. By applying combinations of these neurotransmitters to isolated epithelia from the porcine proximal colon, potentiating effects of ACh and NO and of ACh and VIP on secretion were observed [28]. This finding suggests that ChAT/nNOS-co-localizing neurons probably act as secretomotor neurons, like ChAT/SP neurons.

The small proportion of VIP-immunoreactive neurons in the OSP and the lack of VIP-immunoreactive somata in the ISP are two of the most surprising findings in the present study. These findings differed significantly from the observations of Barbiers et al. (1995) [49] and Balemba et al. (2001) [50], who detected an abundance of VIP-immunoreactive nerve cells and fibres in the ISP, while their numbers were only moderate in the OSP in the porcine colon. The absence of VIPergic neurons in the ISP in our study might be due to the age of the animals examined. Barbiers et al. (1995) and Balemba et al. (2001), observing VIP-immunoreactive neurons mainly in the ISP of the porcine colon, examined pigs at the ages of 18 to 20 weeks and six weeks, respectively. In our study, unweaned piglets with an age of three weeks were studied. Age dependent expression of VIP in enteric neurons was also detected in the porcine cecum. Di Giancamillo et al. (2010) [51] did not find VIPergic neurons in the cecum of three-week-old piglets. In constrast, Balemba et al. (2001) detected an abundant number of VIP-immunoreactive neurons and fibres in the cecum of 18–20-week-old pigs. The assumption that expression of VIP is age dependent in the ISP of piglets is also supported by a study in mice. In the developing murine ENS peptidergic neurons occur later than cholinergic neurons and submucosal neurons develop later than myenteric neurons [52].

Apart from age specific alterations weaning might also influence the development of the submucosal neurons. In piglets, weaning is usually conducted between week three to four. Because of the drastic dietary changes during this time, it is probable that the ENS needs to adapt to new secretory requirements [27, 53, 54]. Therefore, it seems possible that we could not find VIP-immunoreactive neurons in the ISP because they might develop after weaning.

### Neurons immunoreactive for SOM or NPY

In accordance with other studies conducted on the porcine and human colon, the number of SOM-immunoreactive neurons observed was relatively low in both submucous plexuses [55].

Within the gastrointestinal tract, SOM is involved in a variety of processes. SOM inhibits the release of many gut hormones [56] and is involved in the regulation of motility [57, 58], as well as the reduction of blood flow in the gut [59]. SOM also reduces chloride secretion in the porcine jejunum [60] and in the colon of rats [61]. In regard to these various effects, it is surprising that in the porcine small intestine, only one functional class of SOM-immunoreactive neuron has been proposed for both submucous plexuses, namely a class of cholinergic secretomotor neurons [8]. In accordance to the classification of enteric neurons in the porcine small intestine we identified SOM-immunoreactive fibres within the colonic mucosa. Nevertheless, these fibres may not originate only from submucous neurons. In the porcine small intestine Hens et al (2000) observed mucosally projecting, SOM-immunoreactive neurons in the ISP, OSP and MP. Regarding the function of the SOM positive submucous neurons, in the porcine colon a function as secretomotor neurons seems to be excluded by functional studies. Pfannkuche et al. (2011) did not find any evidence for a prosecretory or an anti-secretory effect of SOM in the isolated porcine proximal colon. Therefore, it is reasonable to assume that SOM and SOM-immunoreactive neurons are not involved in the regulation of chloride secretion at least in the proximal colon of pigs. A further possibility for the functional classification of SOM immunoreactive neurons is a function as primary afferent neurons, as suggested for the human colon [62–64]. However, this assumption remains speculative for the porcine colon since a reliable morphological classification of SOM-positive neurons is still lacking.

In the present study, only a low abundance of neurons displaying NPY immunoreactivity was detected in the OSP, suggesting only a minor importance of intrinsic NPY in the porcine colon. The total absence of NPY in the ISP is consistent with the classification of neurons in the porcine small intestine by Brown and Timmermans (2004). Similarly, Pidsudko et al. (2008) did not detect NPY-immunoreactive neurons in the OSP or ISP of the porcine ileum [65]. In contrast, di Giancamillo et al. (2010) found NPY-positive neurons in both submucous plexuses of the ileum, although in the cecum, NPY-immunoreactive neurons were only present in the OSP. Compared to the very low abundance of NPY in the porcine colon, da Silveira et al. (2007) found a huge number of NPY-positive neurons in the human colon, pointing to a species-specific innervation pattern of the colon.

Although only a small population of NPY-immunoreactive neuronal somata was present in the OSP, NPY-positive nerve fibres surrounding the vessels between the ISP and OSP were frequently encountered. Also in cryostat sections, NPY-immunoreactive fibres were present in the mucosa, but without visible connection to the OSP. The latter observation is in accordance with findings of Hens et al. (2000) who could not detect mucosally projecting NPY-immunoreactive neurons in the enteric plexuses of the porcine small intestine. We assume that these NPY-positive fibres probably originate from vasomotor neurons of the inferior mesenteric ganglia or sympathetic chain ganglia [66, 67]. Also it might be speculated that the NPY-immunoreactive neuronal population within the OSP might function as interneurons in the porcine colon, since no NPY positive projections leaving the OSP could be observed in the present study.

### Comparison with human colon

Due to various (patho-)physiologcial similarities, the porcine ENS has been proposed as model of the human ENS which may be even better than the ENS obtained from standard laboratory species [5, 8, 12–15].

Regarding the abundance of the markers and neurotransmitters we evaluated, differences became obvious to exist between pig and human (Table 6). As discussed above, the differences might be due to the variety in the age of the animals/patients examined. It may be that age dependent alterations are larger than interspecies variances. In our study, juvenile pigs around weaning age (21 days old) were used as donor animals. The studies examining the human colon as conducted by Kustermann et al. 2011, Beyer et al. 2013, Beuscher et al. 2014, da Silveira et al. 2007, Anlauf et al. 2003 and summarized in Table 6, used preparations from patients of at least 57 years of age. Age dependent alterations of the neurochemical coding in human enteric neurons have clearly been characterized by Hetz et al. (2014) [68]. The authors observed an increase in gene expression of ChAT and a decrease in nNOS expression in the myenteric plexus of the human colon when comparing children (4month—1 year) with adults (48#x2013;95years). Since detailed studies on the changes in the neurochemical coding in the aging porcine ENS are lacking at present, we are very cautious about recommending the use of porcine intestinal preparations as an alternative model of the human ENS.

**Table 6:** Proportions of neuronal populations in porcine and human colon.

Antigen	porcine SMP^a^		human SMP		
	ISP (%)	OSP (%)	ISP (%)	OSP (%)	SMP (%)
ChAT	42.0	38.3	< 88^b^	< 94^b^	32^e^ - 51^f^
nNOS	0.2	30.2	1^c^	5^c^	10^e^
NPY	0.0	0.2	n.d.	n.d.	30^e^
SOM	0.9	4.3	< 25^b^; 14^c^; 13^d^	< 12^b^; 10^c^; 4^d^	n.d.
SP	29.0	21.1	< 19^b^	< 12^b^	8^e^
VIP	0.1	5.8	77^c^	78^c^	47^e^; 79^f^

^a^ present study, (wholemounts, 3 weeks),

^b^ Beyer et al. 2013 (wholemounts, 66 years),

^c^ Beuscher et al. 2014 (wholemounts, 70 years),

^d^ Kustermann et al. 2011 (wholemounts, 65 years),

^e^ da Silveira et al. 2007 (frozen sections, 57 years),

^f^ Anlauf et al. 2003 (frozen sections, 61 years).

n.d.: not dermined; In brackets: used preparation method and the average age of the examined subjects.

It also should be emphasized, that the preparation method seems to have an impact on the quantification of neuronal populations. Findings derived from frozen sections, like that of da Silveira et al. (2007), Anlauf et al. (2003) (Table 6) or Wedel et al. (2002) differ significantly from those in wholemounts examined by Kustermann et al. 201, Beyer et al. 2013 and Beuscher et al. 2014.

## Conclusions

We found striking differences in the general structure, neuronal density and chemical coding between the ISP and OSP in the proximal colon of three-week-old piglets. A determination of neurochemical subpopulations revealed a strong co-localization of ChAT and SP, which were also the most abundant neuronal markers, in the ISP. Chemical coding in the OSP varied to a higher degree compared to the ISP and was mainly characterized by the presence of nNOS and VIP in addition to ChAT and SP. Fibres origination in OSP ganglia ran mainly within the circular muscle layer or projected to myenteric ganglia. Consequently it can be supposed that the OSP is mainly involved in control of motility processes. In future studies, additional neurotransmitters and neuropeptides should be examined to complete the chemical coding, in particular for cholinergic subpopulations. Furthermore, morphological examinations and age-specific studies would provide valuable insights. The present results suggest that the general architecture and chemical coding of the submucous plexuses in the porcine colon differs in some aspects from that in humans. Therefore, the suitability of porcine colonic innervation as model for the neuronal control of the human colon should be considered with care. Further examination of human tissue is required to assess similarities and differences between the two species. For an accurate quantification of neuronal densities and determination of the neurochemical coding, the preparation of wholemounts is preferred.

## Supporting Information

S1 Appendix Raw Data. (XLSX)

S1 Fig NPY-immunoreactive fibres in the SMP of the porcine colon. Immunohistochemical staining revealed a dense network of NPY-positive nerve fibres surrounding blood vessels between the ISP and OSP. Scale bar = 0.5 mm.
(TIF)

S1 Table *P*-values of significant differences between neurochemically-defined subpopulations in the SMP of the porcine colon. The proportions of subpopulations were compared in the ISP (above diagonal) and OSP (below diagonal) using a two-way repeated-measurements ANOVA, with a subsequent multiple comparison procedure (Student-Newman-Keuls test).
(DOCX)
