# Supplementary figures and images for: Architecture and Chemical Coding of the Inner and Outer Submucous Plexus in the Colon of Piglets
Source: PLoS One. 2015 Jul 31;10(7):e0133350. doi: 10.1371/journal.pone.0133350 (PMC4521800; doi:10.1371/journal.pone.0133350)

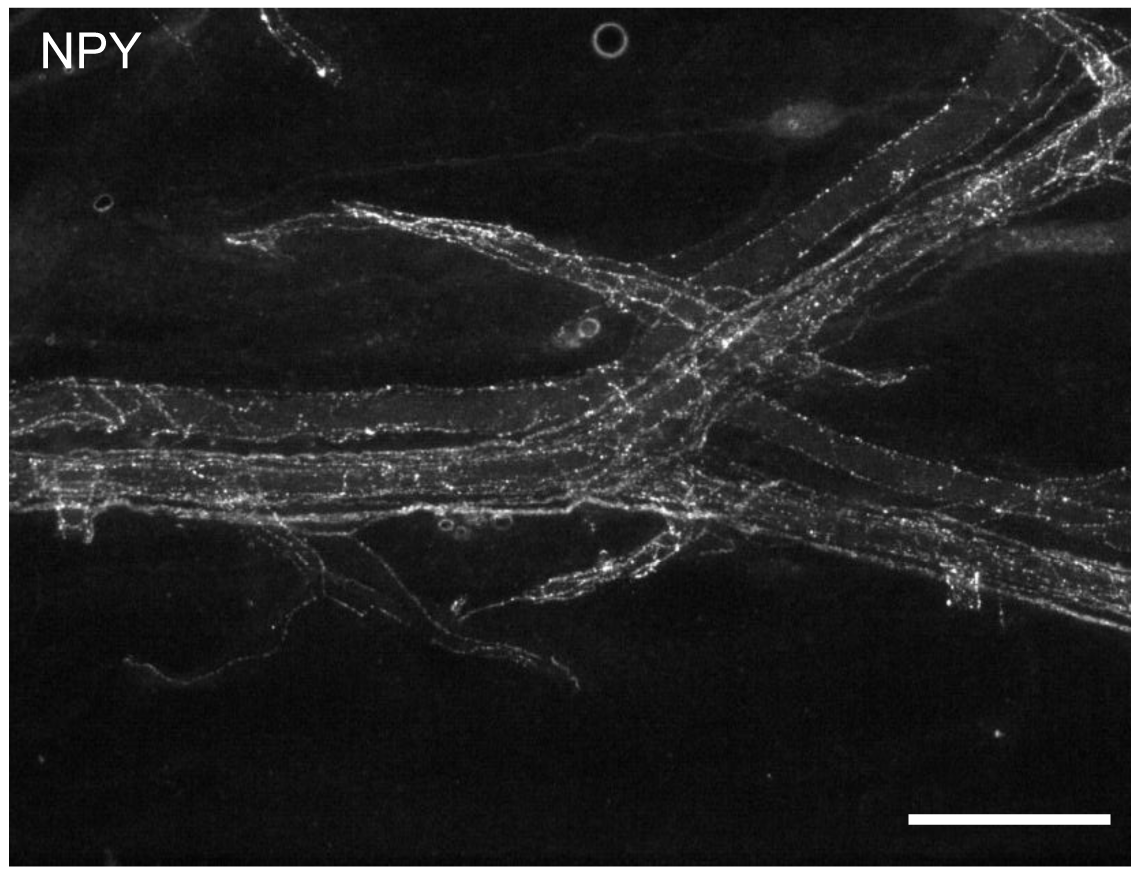

Supplement: S1 Fig — Immunohistochemical staining revealed a dense network of NPY-positive nerve fibres surrounding blood vessels between the ISP and OSP. Scale bar = 0.5 mm. (TIF) [file pone.0133350.s002.tif]
