# Supplementary material for: Architecture and Chemical Coding of the Inner and Outer Submucous Plexus in the Colon of Piglets
Source: PLoS One. 2015 Jul 31;10(7):e0133350. doi: 10.1371/journal.pone.0133350 (PMC4521800; doi:10.1371/journal.pone.0133350)
Supplement: S1 Table — The proportions of subpopulations were compared in the ISP (above diagonal) and OSP (below diagonal) using a two-way repeated-measurements ANOVA, with a subsequent multiple comparison procedure (Student-Newman-Keuls test). (DOCX) [file pone.0133350.s003.docx]

**S2 Table.** ***P*-values of significant differences between neurochemically defined subpopulation in the SMP of the porcine colon.** The proportions of subpopulations were compared in ISP (above diagonal) and OSP (below diagonal) using a two-way repeated-measurements ANOVA with a subsequent multiple comparison procedure (Student-Newman-Keuls test).

| **ISP→**  **OSP↓** |  |  |  |  |  |  |  |  |  |
| --- | --- | --- | --- | --- | --- | --- | --- | --- | --- |
|  | **ChAT/SP** | **ChAT/-** | **nNOS/-** | **ChAT/nNOS** | **NOS/VIP** | **VIP/-** | **ChAT/VIP** | **SP/-** | **ChAT/nNOS/VIP** |
| **ChAT/SP** |  | 0.003 | < 0.001 | < 0.001 | < 0.001 | < 0.001 | < 0.001 | < 0.001 | < 0.001 |
| **ChAT/-** | n. s. |  | 0.002 | 0.002 | 0.002 | 0.002 | 0.002 | 0.002 | 0.002 |
| **nNOS/-** | n. s. | n. s. |  | n. s. | n. s. | n. s. | n. s. | n. s. | n. s. |
| **ChAT/nNOS** | 0.004 | n. s. | < 0.001 |  | n. s. | n. s. | n. s. | n. s. | n. s. |
| **nNOS/VIP** | < 0.001 | 0.018 | < 0.001 | n. s. |  | n. s. | n. s. | n. s. | n. s. |
| **VIP/-** | < 0.001 | 0.005 | < 0.001 | n. s. | n. s. |  | n. s. | n. s. | n. s. |
| **ChAT/VIP** | < 0.001 | 0.004 | < 0.001 | n. s. | n. s. | n. s. |  | n. s. | n. s. |
| **SP/-** | < 0.001 | 0.004 | < 0.001 | n. s. | n. s. | n. s. | n. s. |  | n. s. |
| **ChAT/nNOS/VIP** | < 0.001 | 0.003 | < 0.001 | n. s. | n. s. | n. s. | n. s. | n. s. |  |

n.s. = non-significant
